# Liquid Metal–Polymer Hydrogel Composites for Sustainable Electronics: A Review

**DOI:** 10.3390/molecules30040905

**Published:** 2025-02-15

**Authors:** Abdollah Hajalilou

**Affiliations:** Faculdade de Ciências e Tecnologia, Universidade NOVA de Lisboa (Nova FCT), 2829-516 Caparica, Portugal; e.hajalilou@yahoo.com

**Keywords:** eutectic liquid metal, polymer composite, hydrogel, liquid metal–hydrogel, soft electronics, sustainable materials, wearable applications

## Abstract

Hydrogels, renowned for their hydrophilic and viscoelastic properties, have emerged as key materials for flexible electronics, including electronic skins, wearable devices, and soft sensors. However, the application of pure double network hydrogel-based composites is limited by their poor chemical stability, low mechanical stretchability, and low sensitivity. Recent research has focused on overcoming these limitations by incorporating conductive fillers, such as liquid metals (LMs), into hydrogel matrices or creating continuous conductive paths through LMs within the polymer matrix. LMs, including eutectic gallium and indium (EGaIn) alloys, offer exceptional electromechanical, electrochemical, thermal conductivity, and self-repairing properties, making them ideal candidates for diverse soft electronic applications. The integration of LMs into hydrogels improves conductivity and mechanical performance while addressing the challenges posed by rigid fillers, such as mismatched compliance with the hydrogel matrix. This review explores the incorporation of LMs into hydrogel composites, the challenges faced in achieving optimal dispersion, and the unique functionalities introduced by these composites. We also discuss recent advances in the use of LM droplets for polymerization processes and their applications in various fields, including tissue engineering, wearable devices, biomedical applications, electromagnetic shielding, energy harvesting, and storage. Additionally, 3D-printable hydrogels are highlighted. Despite the promise of LM-based hydrogels, challenges such as macrophase separation, weak interfacial interactions between LMs and polymer networks, and the difficulty of printing LM inks onto hydrogel substrates limit their broader application. However, this review proposes solutions to these challenges.

## 1. Introduction

**Figure 1:**
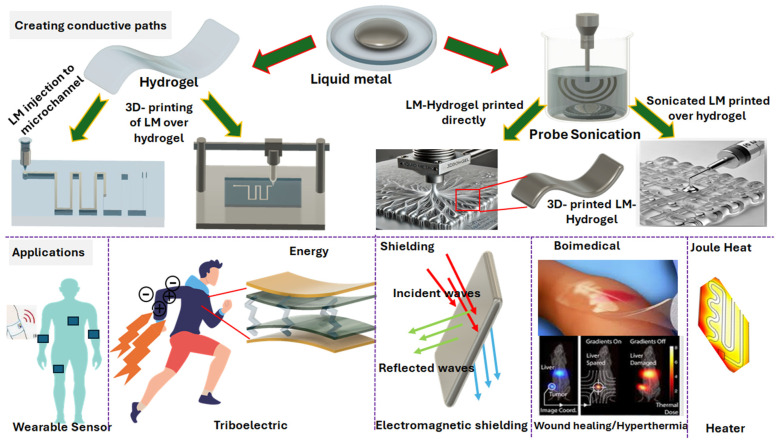
Schematic representation of the LM–hydrogel fabrication process, forming conductive paths, along with their applications.

The inclusion of liquid metals (LMs) into hydrogel composites represents an innovative approach to overcoming the limitations of traditional hydrogel-based soft electronics [1,2,3]. Hydrogels, which are soft materials made of crosslinked polymer networks, exhibit distinct hydrophilic and viscoelastic properties, making them ideal candidates for electronic applications such as electronic skins, wearable electronics, electromagnetic shielding, biomonitoring, energy storage, harvesting, and soft sensors [4,5,6,7,8,9,10,11]. Conductive hydrogels, particularly those with excellent mechanical stretchability and electrical conductivity, are highly desirable for such applications [3,12,13,14,15]. However, hydrogel-based sensors face several challenges, including poor chemical stability, limited mechanical stretchability, low sensitivity, and weak adhesion to human skin [10,16,17]. Addressing these limitations remains a challenge in the field. The choice of conductive fillers is critical for the performance of hydrogel-based electronic devices. Metal nanowires coated on hydrogels can form bendable microelectrodes, but they lack stretchability [18], and conductivity enhancement methods such as sintering are often incompatible with hydrogel substrates. Stainless steel helical springs embedded in hydrogels have been used as wearable force sensors, but the manually twisted patterns limit their design flexibility [19]. Alternatively, ionic hydrogels, prepared by immersing hydrogels in salt solutions, offer a practical approach for constructing circuits [20,21]. While these approaches show promise, they still face issues related to material compatibility, mechanical performance, and scalability. In one approach, these issues can be partially addressed by incorporating rigid conductive fillers like Ag, Cu, and Ni into the hydrogel–polymer matrix [22,23,24], which improves conductivity and sensitivity. Nevertheless, such fillers still pose significant challenges, particularly in terms of durability and stretchability. The rigid nature of these fillers results in a compliance mismatch with the flexible hydrogel matrix, which leads to internal stress concentration, limiting extensibility and causing inelastic stress–strain behavior. This mismatch compromises the long-term durability of the material [25,26]. To overcome these limitations, liquid metal (LM) fillers, such as eutectic gallium and indium (EGaIn) alloys, have recently emerged as promising alternatives in bioelectronic applications [10]. Integrating LM into hydrogel composites offers a novel strategy to address the challenges associated with conventional hydrogel-based soft electronics [1,2,3]. This approach not only addresses the limitations of traditional fillers but also introduces new functionalities into liquid metal-based hydrogel sensors [10,27]. LMs offer several advantages over traditional rigid conductive fillers, including their fluidic nature, high conductivity, excellent ductility, flexibility, non-toxicity, good thermal conductivity, and self-repairing flexibility [19,23,28,29]. LMs can be incorporated into hydrogel composites in two main forms: continuous LM pathways and dispersed LM droplets in a similar manner with their dispersion or injection into the polymer matrix (Figure 1) [23,30,31,32,33], which will be discussed in Section 2. In another approach, the integration of nanoscale colloidal objects into three-dimensional (3D) hydrogel networks can strengthen the hydrogels, acting as “colloidal crosslinkers” while imparting additional functionalities for a wide range of applications [7]. Specifically, combining LM with polymerizable monomers allows for a straightforward one-step procedure to incorporate LM into polymeric systems, resulting in hydrogels with potential applications in tissue engineering, wearable devices, and energy storage devices as solid electrolytes. For instance, one study pioneered the direct polymerization of monomers using LM as an initiator [34]. Recently, LM colloidal droplets have been stabilized as suspensions in both aqueous and organic systems [7,10,30]. These droplets benefit from their inherent fluidic nature and metallic conductivity, making them suitable for applications in microfluidic electronics, printing, and stretchable devices [10,27,35,36]. Moreover, LM droplets have been used as functional fillers to create thermally conductive and dielectric elastomers [30,37,38]. In terms of dispersion, the probe-sonication process has been used to break down LM into micro/nano-sized droplets, which helps to modify the surface tension of the liquid metal. Studies have shown that vinyl-based monomers can stabilize EGaIn liquid metals by interacting with the hydroxyl groups on the surface of EGaIn, thus promoting dispersion and stability [34]. In another study, the role of LM, the type of surfactant, and ultrasonication in LM–hydrogel formation, as well as their electromechanical and electrochemical properties, were systematically investigated [10]. While liquid metal-based hydrogels have shown potential across various applications (Figure 1), as demonstrated in this review, the fabrication of LM–hydrogel nanocomposites faces challenges due to the significant incompatibility between hydrogels and LM, often resulting in macrophase separation [39,40,41]. The weak interfacial interactions between LM and polymer networks result in suboptimal mechanical performance, complicating their integration into high-performance systems. Fully digital printing of LM hydrogels remains challenging. Additionally, directly printing conductive LM composite ink onto hydrogel substrates requires further improvement in ink properties and surface treatment of the hydrogel substrate. To address these challenges, the review identifies innovative material/design modification approaches that could enable the more effective integration of LM composites into hydrogels for next-generation applications. To date, the integration of LM into hydrogel composites has demonstrated promising advancements for flexible electronic applications, offering unique properties like high conductivity, excellent stretchability, and self-healing capabilities. While several studies have explored this area [1,42,43,44,45], the novelty of the current work lies in its deeper exploration of eutectic gallium–indium (EGaIn) alloys within hydrogels, highlighting their potential to overcome the challenges of traditional fillers, such as poor mechanical performance and scalability. This review also focuses on innovative fabrication techniques, such as optimized dispersion strategies and printing technologies, to address material compatibility issues and enhance the performance of LM–hydrogel composites. Furthermore, by exploring emerging applications such as microfluidic electronics, energy storage, electromagnetic interference (EMI) shielding, and multi-sensing devices (Figure 1), this work contributes a forward-looking perspective on the future integration of LM-based hydrogels in next-generation flexible electronics. For instance, future LM-based hydrogel sensors are anticipated to feature self-adhesion, multi-sensing capabilities, low-temperature resistance, re-moldability, and temperature sensitivity to meet the demands of next-generation applications. Moreover, LM–hydrogel composites represent a groundbreaking class of materials for energy applications, uniquely combining the high electrical and thermal conductivity of LM with the flexibility and tunable properties of hydrogels. These composites are driving advancements in energy conversion technologies, particularly in photothermal and thermoelectric systems. By leveraging the synergistic interactions between LM and polymeric matrices, LM–hydrogels exhibit remarkable capabilities in solar energy harvesting and water purification. For instance, their ability to efficiently convert sunlight into heat enables applications in solar-driven evaporation processes and thermoelectric power generation. Additionally, LM–hydrogel composites offer the potential for innovative designs in flexible Joule heating systems and electroluminescent devices, paving the way for sustainable and wearable energy solutions. Despite their promising attributes, challenges such as scalability, environmental stability, and long-term durability must be addressed to unlock their full potential in real-world applications.

## 2. Fabrication Process of LM–Hydrogel Composites

The integration of liquid metals (LMs) with hydrogels has recently gained significant attention. Due to their fluidic nature and high conductivity, LMs can be incorporated into hydrogels in two forms: (i) as continuous pathways and (ii) as dispersed droplets (filler particles) (Figure 1). In the former case, LMs have been successfully embedded into the microchannels of flexible composites [19,43,46]. In the latter case, referred to as colloidal systems, LM droplets are stabilized in organic or aqueous media through sonication, forming highly stable suspensions [10,27]. When nanoscale colloidal LM particles are incorporated into three-dimensional (3D) hydrogel networks, they not only enhance the hydrogel’s strength as “colloidal crosslinkers” but also provide additional functionalities for diverse applications [10].

### 2.1. Conductive Path Through Microfluid Channels in LM–Hydrogel Composites

**Figure 2:**
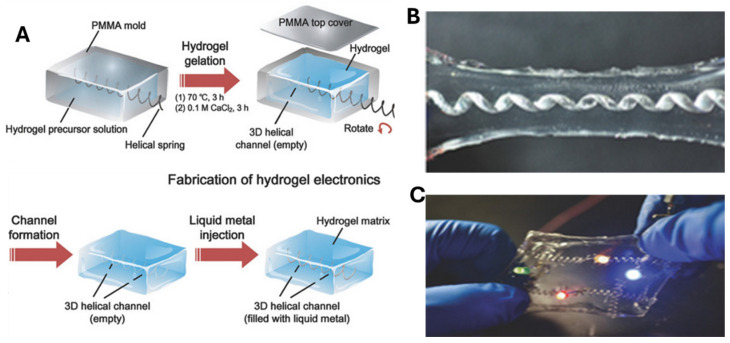
(**A**) Schematic illustration of the fabrication process of hydrogel electronics. (**B**) The LM hydrogel composite was subjected to 150%. (**C**) Fabricated electronic circuit with LM hydrogel composite. Reproduced with permission from [19], Copyright 2018, Wiley.

The microfluidic fabrication process for LM hydrogels involves injecting liquid metal (LM) into the hydrogel. In one study, a PAA–alginate precursor solution was injected into a PMMA casting mold containing a helical spring (Figure 2). The mold was then heated at 70 °C for 3 h to covalently crosslink the PAA network. The mold was immersed in a 0.1 M CaCl_2_ solution for 3 h, facilitating ionic crosslinking of the alginate network to form the PAA–alginate hydrogel. The helical spring was rotated out, creating a 3D microfluidic channel. Liquid metal was injected into the channel, and copper wires were connected for circuit integration. Finally, the hydrogel electronics were sealed with a thin layer of PAA to prevent leakage under mechanical stretching [19]. Table 1 summarizes several examples where the microfluidic approach was employed to fabricate LM hydrogels for various applications. While the microfluidic technique enables the precise and controlled fabrication of LM–hydrogel composites with specific geometries, it may face challenges in scalability for large-volume applications. This is due to the complexity of the design and fabrication process, which requires careful control of variables such as the temperature, pressure, and material properties to maintain the desired structure. Furthermore, microfluidic channels often involve complexity in design and fabrication, which could limit cost-effectiveness in mass production. Despite these challenges, the method offers notable advantages in terms of precision and the ability to produce complex 3D structures. The use of copper wires for circuit integration is a practical solution for embedding electronics, but concerns remain about the long-term stability of the metal interfaces in dynamic, stretchable environments.

Similar to microfluidic systems, Dong Hae Ho et al. introduced an innovative approach to fabricating soft vias and planar interconnects in flexible and stretchable circuits by directing the stratification of LM droplets with programmed photocuring [49]. However, unlike microfluidics, which typically involves transient liquid manipulation, this method creates permanent, conductive pathways within the photoresin. By exploiting UV-induced irregularities at mask edges, the technique forms vertical, stair-like architectures of LM droplets. These droplets settle, assemble, and cure to enable seamless in-plane and through-plane electrical integration. This approach offers tunability, rapid fabrication, and compatibility with a variety of photocurable materials and doped LMs, such as magneto-responsive composites. The resulting devices exhibit high conductivity and mechanical stability without the need for secondary processing steps.

### 2.2. Creating a Conductive Path Involves Mixing or Dispersing LM Filler Particles Within Hydrogel Composites

It has been demonstrated that dispersing conductive rigid filler particles in a polymer matrix improves electrical properties in most cases [50,51,52,53]. However, when these composites are subjected to mechanical stress or strain, they lose their conductivity at relatively high strains due to the mismatch between the filler particles and the matrix. As a solution, liquid metals (LMs), particularly eutectic gallium and indium (EGaIn) alloys, offer better compatibility with stretchable matrices compared to rigid fillers [23,24]. These alloys have shown great promise as soft fillers in hydrogels due to their excellent electrical and thermal conductivity, flexibility, and stretchability [10]. By avoiding stress concentration, LMs enhance the extensibility of the composite material [2,27]. Unlike rigid fillers such as carbon-based materials, and metal particles [54,55,56], LMs offer enhanced mechanical compatibility with hydrogels, reducing internal stress concentration and the risk of puncturing and tearing, which can compromise the mechanical and electrical performance of flexible and wearable sensors [24,30,32]. The unique properties of LMs, including negligible toxicity and high flexibility, make them ideal candidates for soft conductive fillers in epidermal sensors, soft machines, and flexible electronics [23,57,58,59]. When combined with rigid fillers, LM droplets reduce defects in elastic matrices, improving mechanical and electrical properties [23,60,61]. Additionally, LMs undergo redox reactions with substances like oxygen and hydrogen ions, forming compounds such as Ga_2_O_3_, beneficial for LM–hydrogel sensor design [62]. The rupture and reformation of LM oxide layers enable rewritable electronic circuits on hydrogel surfaces, creating flexible, printable, and rewritable sensors [2]. Furthermore, LMs interact with functional groups (e.g., –NH_2_, –OH, –COOH) in hydrogels, resulting in robust composites.

**Figure 3:**
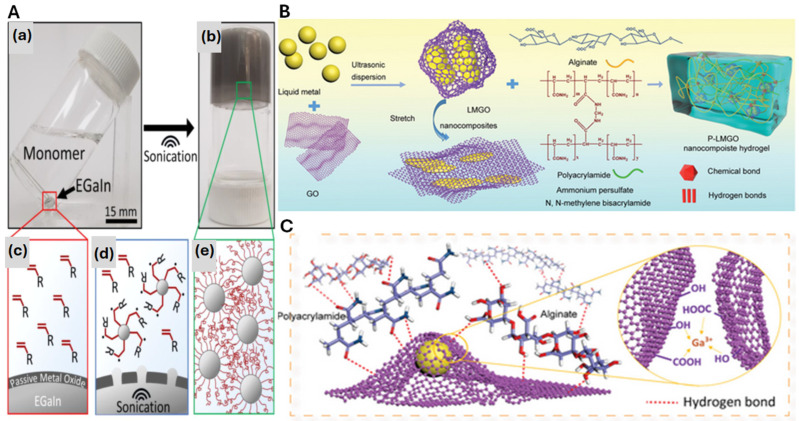
(**A**) Liquid metal particles initiate free radical polymerization without conventional initiators. (**a**) Sonication breaks the oxide-coated metal into smaller particles, exposing the metal to trigger polymerization. (**b**) This forms a physically crosslinked polyacrylamide (PAAm) hydrogel. (**c**–**e**) Schematics illustrate the process. Reproduced with permission from [34], Copyright 2019, ACS publisher. (**B**) Schematic showing the fabrication process of P-LMGO hydrogel with LMGO nanocomposite fillers. (**C**) Interactions between GO, LM, and LMGO nanocomposite fillers with polymers. Reproduced with permission from [68], Copyright 2021, Wiley-VCH.

Despite these advances, LM–hydrogels still require improvements in sensitivity and multifunctionality for complex applications [43]. However, challenges exist in using bulk EGaIn due to its low dispersibility and tendency to aggregate, leading to uncontrollable particle sizes and leakage issues. To address this, the sonication process is employed to disperse micro- or nanostructured LM droplets in the polymer matrix [10], which are stabilized by a thin oxide layer. This process enables the creation of LM particles (LMPs) with size-dependent features such as self-healing, multifunctionality, and stimuli-responsive deformability [63]. Recent techniques, including the use of sulfur polymers, marine polysaccharides, and polyvinyl alcohol (PVA), have been developed to improve the uniform dispersion of LMPs in hydrogels, achieved through surface interactions between the hydroxyl groups of stabilizers and the oxide layer of LMPs [29,64]. This approach represents a critical improvement in LM–hydrogel composites as it enhances the uniformity of LM dispersion and boosts the material’s overall stability. However, it is important to note that the success of these stabilizers heavily relies on the specific conditions of the LM–hydrogel interaction, which can vary depending on the type of stabilizer used and the specific application. The synergistic effect of the oxide layer on LMs and sonication plays a crucial role in enhancing the properties of LM-based hydrogels [10]. Eutectic gallium–indium (EGaIn) and other LMs naturally form a passivating oxide layer, typically Ga_2_O_3_, in ambient conditions, which protects the metal from further reactions [30]. However, this oxide layer can be mechanically disrupted, exposing the metal surface and enabling it to catalyze reactions, such as radical polymerization. This capability allows for the incorporation of LMs into hydrogels through free radical polymerization mechanisms [27,34]. While this disruption and reactivation process is beneficial for enhancing the functionality of LM–hydrogel composites, it also introduces challenges. The reactivity of the exposed LM surface can lead to undesired side reactions or inconsistent polymerization across the hydrogel matrix, affecting the overall performance of the sensor or device. Sonication is commonly used to expose the LM’s surface, where it generates liquid metal nanoparticles (LMNPs) with high surface areas [10]. The mechanical disruption caused by sonication enhances the reactivity of the exposed metal, enabling it to interact with monomers or initiators. These interactions facilitate rapid gelation, as seen in the formation of LM–hydrogels [34]. Research has shown that LMs can directly initiate the polymerization of monomers, either through persulfate radicals or an atom transfer radical polymerization initiator [27]. Additionally, studies demonstrated that the sonication of LM in an aqueous acrylamide solution induces rapid free radical polymerization [34]. Polymerization was initiated by sonicating gallium or eutectic gallium–indium (EGaIn) in an aqueous solution of vinyl-based monomers, such as acrylamide (AAm). Under ambient conditions, both Ga and EGaIn form a passivating oxide layer. Sonication breaks the liquid metal into ~100 nm particles, increasing the surface area to ~50 m^2^/g. Polymerization occurs when Ga or EGaIn is sonicated with vinyl monomers, forming a hydrogel (Figure 3A) [34]. No polymerization happens when the monomer is sonicated alone. The process proceeds similarly even under nitrogen sparging. LMNPs initiate polymerization through a free radical mechanism (Figure 3A(a–e)). The unpaired electron from gallium interacts with the vinyl monomer’s π-bond, forming a covalent bond and generating free radicals that further react with nearby monomers. A critical advantage of this polymerization approach is that it eliminates the need for additional chemical initiators, thus simplifying the process and reducing the number of reagents required. However, while this method is efficient, it may not always provide precise control over the degree of crosslinking, which can be a limitation when designing hydrogels with specific mechanical properties. Furthermore, the polymerization process might be sensitive to variations in temperature, concentration, and monomer types, which can complicate the scaling of this technique for industrial applications. In a study, LM was used as both an initiator and crosslinking agent to create self-healing hydrogels. LM droplets, combined with sonication, polymerized acrylamide (AA) and crosslinked polyacrylamide (PAA) into moldable hydrogels [10]. This “one-step” process expanded LM’s potential by directly polymerizing monomers rather than mechanically shearing polymeric fluids. The LM droplets generated Ga^3+^ ions, forming crosslinked PAA shells that stabilized the droplets. During deformation, the droplets dissipated mechanical energy, imparting high softness, stretchability, and toughness. The PAA network’s breakdown released radicals and Ga^3+^ ions, promoting further polymerization and self-healing. These hydrogels show promise for biomonitoring sensors [7,10]. Additionally, the surface of the LMNPs can be modified by grafting polymers, which influences their dispersion in solvents or water and helps control the interaction between the LM and the hydrogel matrix [65,66,67]. This combination of the oxide layer’s protective role and the exposure of the LM surface via sonication allows LMs to significantly improve the properties of hydrogels, such as mechanical stability, conductivity, and self-healing abilities, making them versatile components for advanced hydrogel applications. Despite the potential of these materials, the grafting of polymers to LMNPs often requires precise control over the grafting density and distribution, as uneven grafting can lead to phase separation or incomplete polymer coverage, which might limit the material’s performance. In one work, P-LMGO hydrogels were created using a polyacrylamide–sodium alginate (PAM–SA) double-network structure, where PAM provided strength and SA maintained integrity. The LMGO suspension was prepared by ultrasonic treatment of graphene oxide (GO) and liquid metal, and the hydrogel was formed by mixing this suspension with SA, PAM, ammonium persulfate (APS), and N,N-methylene bisacrylamide (MBAA) (Figure 3B) [68]. Inspired by biological structures, GO nanosheets with abundant oxygen-containing groups were utilized as “ligaments” to encapsulate eutectic gallium–indium (EGaIn) LMNPs via coordination with Ga^3+^, forming LMGO nanocomposite fillers (Figure 3C). Unlike the poor shape stability of LM droplets and the lack of a bridge between LM and polymers, the GO nanosheet shell enhances the stability of LM droplets and enables them to act as effective crosslinkers. These crosslinkers interact with the polymer matrix through hydrogen and covalent bonds, resulting in a stretchable hydrogel with high elongation and toughness. The LMGO also imparts notch insensitivity and strong adhesion to the hydrogel. Additionally, the oxidation of liquid metal accelerates hydrogel formation at room temperature by releasing free radicals. Combined with shear-thinning behavior, these properties make the hydrogel a promising material for wearable next-generation electronics.

In another study, LM–hydrogels were prepared via in situ polymerization, where liquid gallium was dispersed into a monomer solution using 5 min of sonication. The LM droplets remained stable for over 60 min in the polar monomer solution (acrylamide and 2-hydroxyethyl acrylate) due to surface interactions between the polar groups (–NH_2_, –OH) and the GaO_2_ oxide layer [39,40,41]. In contrast, LM droplets precipitated from pure water within three minutes. After achieving stable dispersion, gelation occurred within 20 s upon adding ammonium persulfate (APS). Without LM, the monomer solution did not gel, suggesting that LM promotes radical formation. Iodometric testing showed that APS with LM only partially oxidized potassium iodide (KI), indicating LM acts as a redox catalyst, similar to other reducing metals [69,70]. LM’s catalytic and surface anchoring effects facilitated rapid gelation and prevented the significant aggregation of LM droplets. Samples with varying LM concentrations (0 vol%, 5 vol%, 10 vol%, and 20 vol%) maintained uniform dispersion, with droplet sizes of 5–8 µm, though aggregation occurred at 20 vol% LM [27]. Another study utilizes the properties of Ga LM to trigger both the polymerization of poly(acrylic acid) (PAA) and the deoxygenation of graphene oxide (GO) to form reduced graphene oxide (rGO), which leads to the formation of conductive networks and porous structures in the hydrogel [43]. The process begins with acrylic acid (AA) as a reactive monomer, which creates an acidic environment due to the ionization of the –COOH groups. This acidic condition enables Ga LM to trigger the deoxygenation of graphene oxide (GO) to form reduced graphene oxide (rGO), resulting in an electronically conductive LM–rGO hybrid network. Simultaneously, Ga LM induces the polymerization of poly(acrylic acid) (PAA) in the presence of ammonium persulfate (APS), with the reaction between Ga LM and H^+^ producing hydrogen bubbles and Ga^3+^ ions. The Ga^3+^ ions interact with the –COO^−^ groups in PAA, forming a supramolecular hydrogel network that incorporates the hydrogen bubbles, thus creating microporous structures within the hydrogel matrix. Ga LM acts both as a soft conductive filler and as an inducer of porous hydrogel formation, resulting in composite hydrogels with high stretchability, elasticity, and self-healing properties. This method enables the creation of hydrogels with high compressive sensitivity, strain sensitivity, and other multisensory capabilities, such as temperature, solvent, and vacuum sensitivity. The resulting hydrogels show great potential for applications in artificial flexible devices, particularly as stress–strain sensors [43].

## 3. Three-Dimensional Printable LM Hydrogels

**Figure 4:**
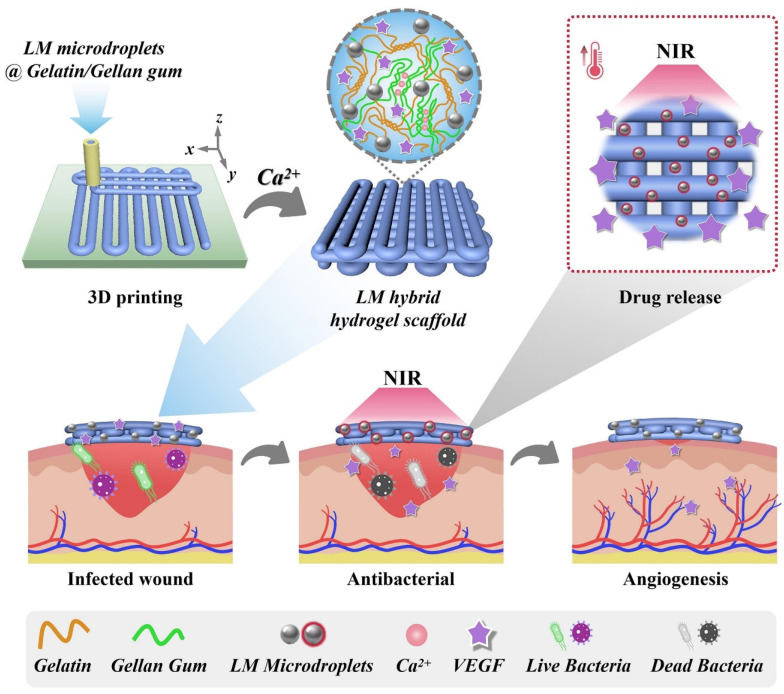
Schematic illustration of the fabrication process of LM hybrid hydrogel scaffolds via 3D printing and their application in infected wound treatment. Reproduced with permission from [77], Copyright 2024, Elsevier.

Three-dimensional printing is an additive manufacturing technique that enables the creation of complex three-dimensional structures layer by layer using computer-aided design. Since its inception as stereolithography in 1981, and the development of fused deposition modeling (FDM) in 1992, 3D printing has revolutionized various fields, including aerospace, architecture, and tissue engineering (TE) [71]. Three-dimensional bioprinting uses bioinks, made of biomaterials and living cells, to create patient-specific tissues and hybrid structures that promote cell growth and repair. Applications like artificial nerve conduits and connective tissues show promise in regenerative medicine. Choosing appropriate hydrogels for bioinks is crucial for successful constructs, as they must meet specific requirements for both printing and tissue engineering [72,73,74,75]. Recently, the integration of LMs with hydrogels has emerged as a transformative innovation, particularly in the domain of flexible electronics [10], which offers a significant step forward in creating flexible, multifunctional materials for electronics. Three-dimensional printable LM hydrogels combine liquid metal’s conductivity and flexibility, enabling intricate, rewritable, and reconfigurable circuits resettable via mechanical scratching and hydrogel swelling. These hydrogels offer the potential for applications in flexible displays, wearable technologies, sensors, and smart surfaces, paving the way for sustainable, adaptive electronic devices [2,76,77,78,79]. However, the fabrication of macroscopic 3D structures with LM hydrogels presents several technical hurdles. One of the most notable challenges is the instability of liquid metal in suspension, which can be exacerbated by fluid dynamics, gravity, and surface tension during the 3D-printing process. Suspension 3D printing employs self-healing hydrogel supports to counteract these issues, but there remains a critical need for further refinement in the control of printing parameters such as speed, nozzle diameter, and gel concentration. These factors directly influence the deposition of liquid metal droplets into intricate patterns, affecting both the microstructure and functionality of the printed devices [76]. A 3D printable LM hydrogel, using eutectic gallium–indium (EGaIn) particles dispersed in poly(ethylene glycol) diacrylate (PEGDA), enables rewritable and printable electrical circuits. The surface can be scratched to expose the conductive EGaIn and erased by swelling the PEGDA, resetting the circuit. The hydrogel demonstrated excellent writing–erasing endurance over 20 cycles, with electrical resistance transitioning from ~1 Ω (conductive) to ~10^7^ Ω (insulating). This feature of rewritability and high endurance is one of the significant advantages of LM-based 3D printable hydrogels over traditional printed circuits, which often fail after repeated use or mechanical deformation. Using surface friction pen printing, flexible and rewritable electrical conductors were successfully created, showing potential for applications like displays [2]. Despite these strengths, the primary limitation of this system is the mechanical instability of the hydrogel matrix when subjected to stress or external environmental factors. Although the hydrogel’s flexibility is improved by incorporating LM droplets, the matrix itself must be designed to withstand the wear and tear associated with long-term use. Additionally, while PEGDA-based systems are promising for 3D printing, their reliance on swelling for circuit resetting may introduce limitations in device lifespan and reliability, especially under continuous deformation. In another study, to prepare hydrogel ink with LM microdroplets, a gelatin solution was mixed with LM bulk and sonicated in an ice bath to form the LM–gelatin mixture. This mixture was combined with gelatin and gellan gum solutions to create the LM hybrid hydrogel ink. The ink was 3D-printed at room temperature, and after printing, the scaffold was soaked in CaCl_2_ solution for ion crosslinking to form the final LM hybrid hydrogel scaffold (Figure 4) [77]. The ionic crosslinking reaction between gellan gum with Ca^2+^ enhanced the stability of the printed LM hybrid scaffolds . The use of gellan gum and gelatin as hydrogel components brings advantages in biocompatibility and ease of crosslinking, enhancing the structural integrity of the printed scaffolds. However, the mechanical properties of these materials may still fall short of the durability required for long-term, robust applications in flexible electronics. LM’s photothermal responsiveness enabled controllable antibacterial effects and drug release, promoting wound closure and angiogenesis. VEGF incorporation ensured sustained angiogenesis. The multifunctional nature of these hydrogels, combining antibacterial, drug release, and angiogenesis promotion, is one of the standout features, making them suitable for a range of biomedical applications. In vivo studies confirmed the scaffolds’ efficacy in wound healing. These findings highlight the potential of LM hybrid hydrogel scaffolds for wound treatment and biomedical applications, with further research needed for deeper insights.

**Figure 5:**
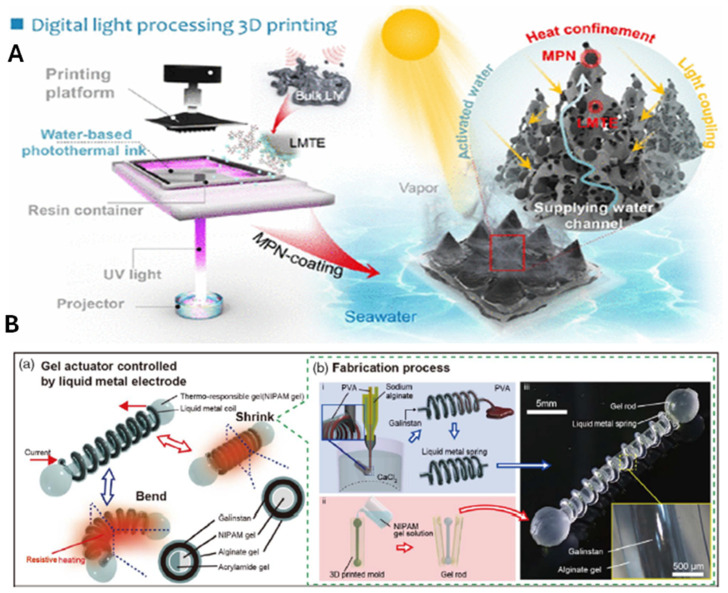
(**A**) 3D-printed hydrogel milli-conical needle arrays for solar steam generation: hydrogel with a 3D conical needle architecture for omnidirectional solar harvesting and enhanced performance. Reproduced with permission from [79], Copyright 2024, ACS Publications. (**B**) Hydrogel actuator with a PNIPAM rod and liquid metal spring. (**a**) Resistive heating from the spring controls rod shrinking or bending. (**b**) The spring is fabricated using bevel-tip nozzle techniques, and the rod is molded with a 3D-printed mold. Reproduced with permission from [80], Copyright 2020, Wiley-VCH.

In another study, a 3D printable PxLMTEi@MPN hydrogel was developed using a photo-crosslinking technique with water-based photothermal inks and LM nanodroplets (Figure 5A) [79]. The fabrication process included 3D printing, freeze-drying, and functionalization with metal–phenolic networks (MPNs) to enhance light absorption and photothermal properties. The incorporation of AGE-modified LMTEi nanodroplets improved the UV response, broadband light absorption, and photothermal conversion efficiency (67.5%). The optimized hydrogel evaporator (P0.25LMTE6@MPN-5) featured a milli-conical needle array morphology and demonstrated exceptional performance, achieving a high evaporation rate of 2.96 kg m^−2^ h^−1^ and energy efficiency of 96.93% under 1 sun irradiation. Outdoor solar desalination tests showed stable evaporation rates (2.35–2.73 kg m^−2^ h^−1^) with desalinated water suitable for crop cultivation without secondary pollution. This approach highlights the potential of DLP 3D printing and metal–phenolic coordination chemistry for designing advanced solar evaporators [79]. In another study, a 3D printable self-healing hydrogel was used for suspension printing of liquid metal structures. Carbopol 940 was dispersed in deionized water, pH adjusted to 7.5, and degassed. Printing parameters like nozzle diameter, flow rate, and speed controlled droplet size and spacing. This method enabled complex 3D liquid metal structures, overcoming fluid instability and surface tension, with the potential for flexible electronics [76]. In one study, a thermoresponsive gel actuator was developed with LM as the thermal stimulator. An LM hydrogel spring surrounds the PNIPAM rod, which undergoes volume changes with temperature fluctuations. Above its LCST, PNIPAM releases water, and below it, it absorbs water. The liquid metal’s resistive heating drives rod shrinkage, enabling bending motion through the bilayer structure of non-thermoresponsive and thermoresponsive gels (Figure 5B(a)) [80]. Electricity controls its actuation, simplifying its operation. The soft, stretchable hydrogel spring enables unhindered actuator movement. Feasibility is demonstrated through expansion, contraction, and bending experiments. The LM spring was fabricated using the bevel-tip and double bevel-tip nozzle methods for the spring and core–shell structures, respectively (Figure 5B(b(i))) [81]. A helical hydrogel structure formed due to crosslinking imbalances from the nozzle’s inclination. A core–shell structure was created using double nozzles, with PVA as the core and sodium alginate as the shell. Galinstan LM was injected into the PVA solution, while a PNIPAM-based hydrogel rod provided actuation. The pregel solution was molded, and the rod detached (Figure 5B(b(ii))). PNIPAM polymerized onto the alginate gel to form a bilayer structure, and the liquid metal spring and thermoresponsive hydrogel rod were integrated to create the actuator.

The studies exemplified above indicate that integrating 3D-printing technologies with LM hydrogels represents a significant leap forward in both electronics and biomedicine. By combining the versatility of 3D printing with the unique properties of liquid metals, these systems have the potential to drive innovation across diverse fields, paving the way for the next generation of sustainable, functional, and adaptive materials and devices. However, the method requires further optimization in terms of material stability, uniformity and scalability with fabrication precision, and the long-term durability of the structures.

## 4. Categorization of Liquid Metal Integration in Hydrogels and the Role of 3D Printing

As discussed in Section 2 and Section 3, the integration of liquid metals (LMs) into hydrogels is primarily categorized into two distinct forms: (i) continuous LM pathways and (ii) dispersed LM droplets. These approaches define the structural and functional nature of LM–hydrogel composites. Continuous LM pathways involve embedding LMs as interconnected conductive networks within the hydrogel matrix, forming macroscopic paths that enable high electrical conductivity. This is typically achieved by injecting or embedding LM into pre-formed microchannels or directly printing LM onto a hydrogel substrate, resulting in composites with excellent electrical performance and macroscopic structural integrity. On the other hand, dispersed LM droplets are created by sonicating LM into micro- or nanoscale droplets, which are then stabilized in aqueous or organic media (Figure 1). These droplets act as “colloidal crosslinkers” when incorporated into hydrogels, enhancing mechanical properties and introducing additional functionalities, such as improved flexibility and localized conductivity.

The development of 3D-printable LM hydrogels, while leveraging these established forms of LM integration, requires careful categorization to avoid misinterpretation as a separate or third category. Specifically, when LM is sonicated into droplets and incorporated into a polymer matrix during the 3D-printing process, the resulting material aligns with the dispersed LM droplet category. The printing process enables precise spatial distribution of LM droplets within the hydrogel, offering tailored mechanical and conductive properties, as presented in Ref. [10]. Conversely, when LM is directly printed onto a hydrogel matrix, this fabrication technique corresponds to the continuous LM pathway category. This method facilitates the creation of well-defined conductive networks, such as circuits or microchannels, integrated into the flexible hydrogel structure, as can be seen in Ref. [8].

Although 3D-printable LM hydrogels represent a significant advancement in fabrication techniques, they should not be regarded as a third form of LM integration. Instead, they are extensions of the two primary forms, with their classification determined by whether the LM is utilized as dispersed droplets or as a continuous conductive pathway. By maintaining this distinction, the versatility of LM–hydrogel systems can be effectively highlighted while ensuring clarity and consistency in their categorization.

## 5. Printing of the LM-Based Inks over Hydrogel Substrate (Interface Engineering)

Typically, EGaIn particles are embedded in single layers and shear-mixed with elastomeric polymers. This approach results in deformable electrodes that are challenging to print directly onto substrates, limiting their application in deformable microelectronics [82]. On the other hand, soft, biocompatible hydrogels are ideal for stretchable electronics but often face issues like poor conductivity and mechanical mismatch with conductive materials. This suggests that the direct patterning of nontoxic, highly conductive liquid metal (LM) onto hydrogels, addressing these challenges but spreading liquid droplets on solid surfaces, is a key topic in physical chemistry with important technological implications [83,84]. Liquid metals, such as eutectic alloys with low melting points, offer practical utility but are challenging to spread on solid surfaces due to their high surface tension. This presents a barrier to using LMs as deformable electrodes in on-board microcircuits, despite their inherent deformability. A study found that eutectic gallium–indium (EGaIn) can be spread onto chemically crosslinked hydrogels with aliphatic alkyl chains and hydroxyl groups (–OH), enabling micropatterned EGaIn electrodes. EGaIn autonomously restores its surface to form a strong interface when the hydrogel deforms. This self-healing feature, combined with the hydrogel’s reversible stretching, self-healing, and water-swelling properties, leads to superstretchable, self-healable, and water-swellable liquid-metal electrodes with high conductance tolerance, making them ideal for deformable microelectronic applications [85]. Three-dimensional printing of LMs on hydrogels has emerged as a promising approach, but challenges remain due to the high surface energy and low viscosity of LM, which makes it non-adhesive to the hydrogel surface. Although the rapid formation of an oxide layer on LM particles creates a stabilizing layer that aids in 3D printing, printability remains a challenge. For instance, researchers have used self-healing Carbopol hydrogels as a supportive medium, allowing LMs to be printed into arbitrary macrostructures. Key parameters such as nozzle diameter, flow rate, and printing speed control the size and precision of the printed LM droplets [76]. While Carbopol hydrogels provide good printability, they still cannot fully address the intrinsic adhesion problem between LM and hydrogels, which remains a critical barrier to achieving precision printing. Another approach employs a suspension printing method, where Galinstan is extruded into continuous filaments with a spatial resolution of 150 μm. These filaments are deposited as 3D freeform structures within an acrylamide (AAm)–nano-clay suspension, which serves as the support bath. After deposition, the AAm–nano-clay suspension is cured into a springy hydrogel, encapsulating the LM filaments [86]. This method shows promise in overcoming some of the adhesion challenges by encapsulating the LM filaments, which ensures a more stable interface and offers improved printability. However, the need for an additional curing step can increase complexity and cost, which may hinder its scalability for large-scale production.

**Figure 6:**
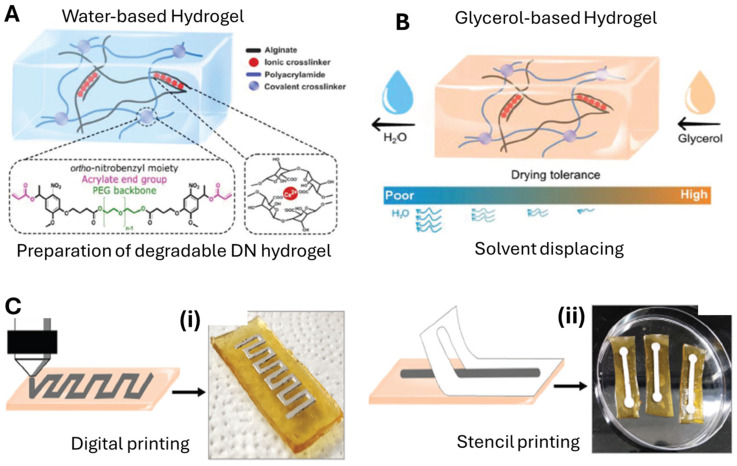
(**A**) Alginate–PAAm hydrogel structure with ONB-PEG600 covalent crosslinking and Ca^2+^ ionic alginate crosslinking. (**B**) Enhanced drying tolerance via in situ water-to-glycerol solvent displacement. (**C**) Digital and stencil printing of the LM-based ink over hydrogel substrate, where (**i**), and (**ii**) show the digital printing, and stencil printing of the composite ink over hydrogel. Reproduced with permission from [8], Copyright 2023, Wiley.

In one study, a hybrid double-network (DN) photodegradable alginate–polyacrylamide (PAAm) hydrogel—where alginate was ionically crosslinked with divalent calcium ions (Ca^2+^), and PAAm was covalently crosslinked using a synthesized o-nitrobenzyl (ONB)-based crosslinker—has been developed for bioelectronics, offering a combination of excellent mechanical properties, non-drying capability, and photodegradability. Soft-matter circuits were fabricated using a biphasic liquid metal-based ink [23]. A liquid metal-based biphasic ink was developed by incorporating GaIn liquid metal and Ag-flake filler particles into a thermoplastic styrene–isoprene–styrene (SIS) elastomer matrix, which could be efficiently printed on various substrates. The preliminary results indicated that the LM-based ink, specifically liquid metal (EGaIn)–Ag flake–SIS [23,28,32], which has shown adhesion to various substrates, does not adhere or stick to the as-prepared hydrogel (Figure 6A,B) [8]. The development of biphasic ink has the potential for widespread application due to its versatile adhesion properties, but the lack of adhesion to the hydrogel limits its immediate applicability in the field. This suggests the need for further optimization of the ink composition to achieve stronger bonding with hydrogel substrates. Circuit patterns were fabricated using both digital and stencil printing techniques. Initial attempts to print on water-based hydrogels failed due to cracking, likely caused by the high water content interfering with ink drying. In contrast, glycerol-based hydrogels improved ink adhesion, attributed to the formation of gallium oxide hydroxide (GaOOH) and Ga_2_O_3_ crystallites that bond with glycerol’s hydroxyl groups. This enabled successful printing using both methods (Figure 6C(i,ii)). The use of glycerol-based hydrogels represents a promising solution to the adhesion issue, as it facilitates better bonding between the ink and the substrate, although the choice of hydrogel must be carefully matched to the LM ink’s properties to avoid cracking and drying issues. To evaluate electromechanical performance, the ink was stencil-printed onto rectangular hydrogel substrates. Electrical resistance measurements revealed a minimal resistance increase during cycling, remaining below 2 Ω, suitable for digital circuits. Notably, in the first strain cycle, the resistance significantly decreased (e.g., from ~2.5 Ω to 0.6 Ω at 100% strain), attributed to the mechanical breakdown of the oxide shell on liquid metal droplets, enhancing percolation. These results demonstrate excellent electromechanical stability and good adhesion between the ink and the hydrogel substrate. The performance is comparable to previous studies using elastic latex substrates, highlighting the hydrogel’s elastic properties and suitability for advanced printing applications [8]. While the electromechanical performance shows promising stability and low resistance, the mechanical breakdown of the oxide shell under strain may limit the longevity of the printed circuits, suggesting a trade-off between short-term performance and long-term durability.

In another study, patterning is achieved using magnetic microparticles and a shadow mask created by in situ laser cutting. The resulting LM-based hydrogel electronics show mechanical and electrical self-healing properties and are applied in wearable sensing, wireless communication, and soft actuators. The high-resolution LM patterning technique for hydrogels offers several key advantages: it is simple, fast, and requires no pretreatment of the hydrogels; the noncontact laser cutting and magnetic coating steps prevent the mechanical deformation of the soft hydrogels; and it is cost-effective, making it suitable for large-scale production [87]. However, the noncontact nature of the laser cutting process can sometimes lead to limited resolution or precision, particularly for complex microstructures. Furthermore, the use of magnetic microparticles introduces potential challenges in controlling the particle alignment and distribution, which could impact the final performance of the patterned LM-based structures. In one study, researchers developed a novel approach for metalizing transparent hydrogel atomic force microscopy (AFM) cantilevers using eutectic gallium–indium (EGaIn) as a reflective coating. This method addressed the limitations of traditional metallization techniques, such as sputtering or evaporation, which are time-intensive and require cleanroom facilities. By applying oxygen plasma treatment to the poly(ethylene) glycol-diacrylate (PEG-DA) hydrogel surface, they enhanced the adhesion and uniformity of the EGaIn coating. The resulting ~800 nm thin reflective layer enabled the hydrogel cantilevers to function effectively with optical-lever detection systems, demonstrating high performance in AFM imaging in both air and deionized water environments [88]. While this approach provides a significant advantage over traditional methods in terms of time and facility requirements, the thinness of the EGaIn coating may limit its mechanical robustness, especially under dynamic conditions. Furthermore, the plasma treatment step introduces additional complexity, which could affect the scalability and reproducibility of the process.

## 6. Liquid Metal Hydrogels Applications

### 6.1. LM–Hydrogel Composite in Electromagnetic Interference (EMI) Shielding Applications

**Figure 7:**
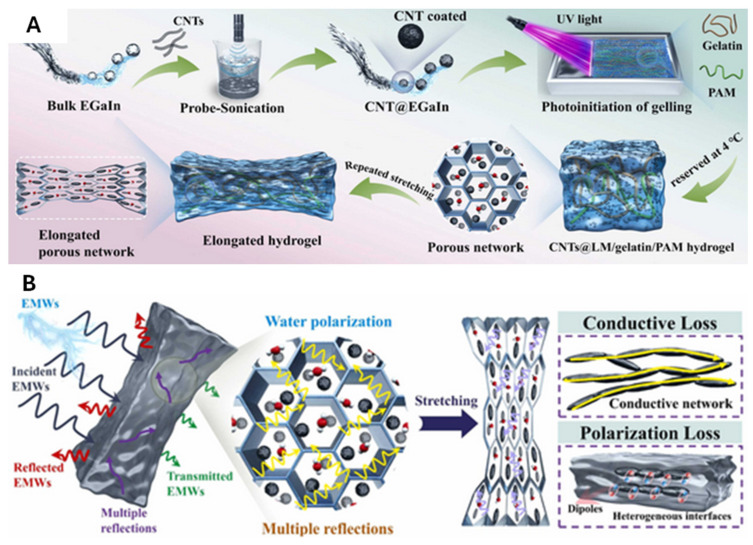

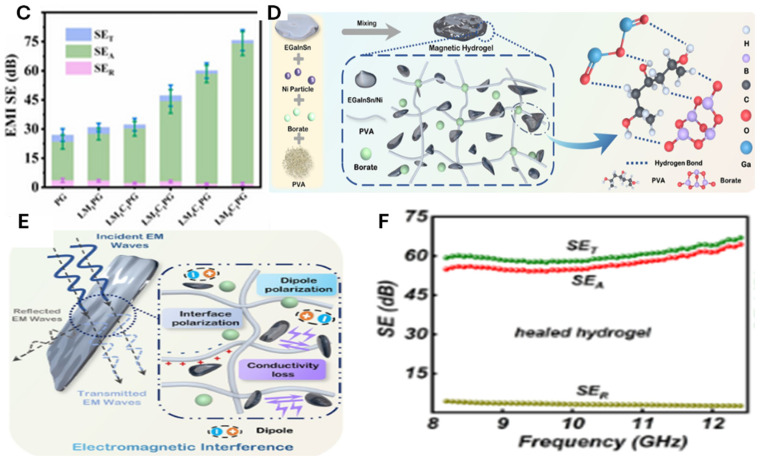
(**A**) Schematic illustration of the fabrication process for the LMCPG hydrogel, (**B**) EMI shielding mechanism, and (**C**) EMI shielding performance. Reproduced with permission from [90], Copyright 2023, Elsevier. (**D**) Schematic preparation of the PVA–EGaInSn–Ni composite hydrogel with primary crosslinked networks. (**E**) EMI shielding mechanism of the LM-based hydrogel, attributed to conductive loss, interfacial polarization, and dipole polarization. (**F**) EMI shielding performance. Reproduced with permission from [95], Copyright 2023, Springer.

Liquid metal (LM) hydrogels have emerged as promising materials for electromagnetic interference (EMI) shielding applications, addressing the limitations of existing stretchable conductive materials [89,90,91,92]. Traditional stretchable materials often experience a significant decay in EMI shielding performance under mechanical deformation, such as stretching, which restricts their practical usability [93,94]. Moreover, designing materials with ordered porous frameworks for integrated EMI shielding remains a significant challenge due to the complexity of fabricating structures that enhance multiple reflections of electromagnetic waves (EMWs). In this context, LM hydrogels play a crucial role by combining stretchability, durability, and high shielding efficiency. However, the reliability of these materials under prolonged mechanical deformation and environmental factors such as temperature and humidity still require further evaluation to confirm their long-term stability. In one study, an LM–CNT–gelatin–PAM hydrogel composite was developed for EMI shielding applications. The fabrication of the LM-based double-network hydrogel (CNT@LM–polyacrylamide–gelatin or LMCPG) involved ultrasonic agitation to create stable CNT@LM droplets (Figure 7A) [90]. High-power ultrasound broke the LM into small droplets coated with Ga^3+^ cations, which interacted with functionalized CNTs to form a stable colloidal suspension. The hydrogel, produced via UV in situ polymerization, exhibited a dual-network structure: a rigid network for strength and a flexible one for adaptability, achieving a tensile strength of 117 kPa at 1033% strain. Under stretching, the deformable CNT@LM droplets elongated, aligning randomly to form additional conductive pathways, resulting in a 211% increase in EMI shielding effectiveness (SE) at 200% strain. The porous hydrogel framework enhanced shielding by enabling multiple EMW reflections and absorptions. Mechanisms like impedance mismatch, polarization loss from water dipoles, and interfacial polarization between LM and the hydrogel matrix further dissipated the EMW energy (Figure 7B). The EMI shielding performance of the LM–hydrogel is given in Figure 7C at about 75 dB. This intelligent shielding behavior was demonstrated in a wireless power-transfer system, where the hydrogel disrupted resonance and extinguished light at 0% and 200% strain, highlighting its practical potential. While this approach demonstrates excellent stretchability and shielding effectiveness, the reliance on high-power ultrasound for creating stable colloidal suspensions could limit scalability and introduce variations in the droplet size distribution. Additionally, the long-term stability of the conductive networks formed under such strain conditions must be thoroughly investigated.

In another study, self-healing PVA-based hydrogels were fabricated by dissolving PVA in deionized water and incorporating EGaInSn and Ni particles through ultrasonic treatment. Sodium tetraborate was added to form composite hydrogels with varying EGaInSn–Ni mass ratios [95]. Figure 7D illustrates the fabrication process, where ultrasonic treatment produced EGaInSn suspension, which was then mixed with Ni, PVA, and sodium tetraborate. Borate molecules served as crosslinkers by forming hydrogen bonds with PVA, while the oxide layer on the liquid metal provided additional crosslinking with PVA chains. As shown in Figure 7E, conductive networks formed by LM droplets, water dipoles, and interfaces between PVA, EGaInSn, and Ni was integral to the hydrogel’s performance. Stretching caused LM droplets to break and reconnect, creating conductive pathways that enhanced EMI shielding [30]. The hydrogels exhibited a total shielding effectiveness (SE) of 65.8 dB, an 83% improvement over pure PVA hydrogels (Figure 7F). The incorporation of self-healing and crosslinking strategies significantly enhanced the material’s ability to maintain EMI shielding efficiency under mechanical deformation. However, the reliance on multiple components like sodium tetraborate and Ni particles could complicate the fabrication process and limit the homogeneity of the final product. Simplifying the fabrication process while maintaining or improving performance remains a challenge for future studies.

### 6.2. LM–Hydrogel Composite in Energy Applications

**Figure 8:**
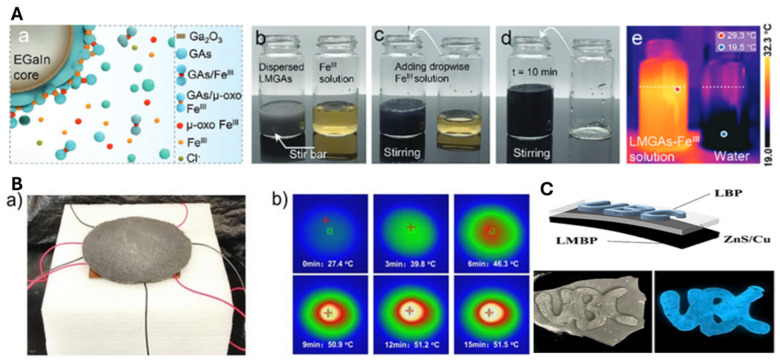
(**A**) Schematic of the rapid assembly process of LMGAs-FeIII, highlighting interface and bulk species formation (**a**). Digital images showing the progression of the assembly in the LMGAs dispersion (**b**–**d**). IR images of the stable temperature of LMGAs-FeIII solution and DI water under one sun irradiation (**e**). Reproduced with permission from [100], Copyright 2023, Wiley. (**B**) Thermoelectric performance of the EGaIn@Ag–PVAG hydrogel: (**a**) Photograph of the SDTC device; (**b**) Infrared thermal images and temperature increase profile of the hydrogel under solar irradiation. Reproduced with permission from [98], Copyright 2024, Elsevier. (**C**) Patternable electroluminescent devices. Reproduced with permission from [101], Copyright 2023, Elsevier.

Liquid metal (LM) hydrogels have gained significant attention in energy applications due to their unique combination of excellent electrical and thermal conductivity, enabling efficient energy conversion across various technologies [68,96,97,98]. The ability of these composites to efficiently harness energy in different forms positions them as promising candidates for advancing sustainable energy technologies such as photothermal conversion, electroluminescence, and electrothermal systems. However, further studies on their scalability, energy conversion efficiency, and long-term operational stability are crucial for practical implementation. One of the most promising energy conversion capabilities of LM–hydrogel composites is in photothermal conversion, which allows for effective solar energy utilization. By absorbing solar radiation and converting it into heat, these hydrogels can serve in solar-driven evaporation processes. For example, the integration of LM with polyaniline (PANI) enhances the photothermal conversion efficiency through a synergistic interaction between the free electron oscillations of LM and the lattice vibrations of PANI. This combination shows great potential for applications such as water purification, where the composite hydrogel can efficiently convert sunlight into heat to drive evaporation [99]. However, potential issues with the long-term stability of the composite, in solar energy absorption applications, in varying environmental conditions (e.g., humidity and temperature fluctuations) need further attention. In another study, photothermal hydrogels with broadband light absorption and highly hydrated networks offer an efficient platform for solar-driven water evaporation. By integrating spectrum-tailored liquid metal droplets (LMGAs-FeIII) and carbon-wrapped silver nanowire sponges (Ag@C 750) into a poly(vinyl alcohol) hydrogel (PALGH), a dual-mechanism vaporization structure is created, enhancing heat confinement and light-to-heat conversion. Under one sun irradiation, PALGH achieves a brine evaporation rate of 3.47 kg m^−2^ h^−1^, producing over 19 L of clean water daily, demonstrating its potential for seawater purification. This work offers a design principle for advanced photothermal materials and solar heat generation in cross-media systems. Figure 8A(a–e) illustrate the fabrication of LMGAs-FeIII, where bulk LM is sonicated in a GA solution to form LMs wrapped in GA/GAs molecules, turning dark gray after centrifugation. High-frequency sonication triggers radical-mediated C–C coupling, forming oligomeric phenolic species and ellagic acid (EA). EA then reacts with GA/GAs to create phenol-based derivatives. Adding FeCl_3_ induces coordination between GAs and FeIII, forming chelate complexes that stabilize the LM interface, resulting in a dark blue suspension [100]. This advanced approach shows great promise in solar desalination, but the complexity of the fabrication process, including the use of multiple chemical components, may limit large-scale applications. Optimization for cost-effective, scalable production is an important area for future work.

LM–hydrogels are also utilized in thermoelectric power generation, where they absorb solar energy, convert it into heat, and create a temperature gradient that can be harnessed to produce electricity through thermoelectric devices. This process can enable sustainable and cyclic power generation, making these composites suitable for energy harvesting applications. One study developed a photothermal EGaIn@Ag–PVAG hydrogel composite, to enhance solar-driven evaporation (SDIE) and thermochemical conversion (SDTC) [98]. The design incorporates EGaIn@Ag nanoparticles for efficient photothermal conversion, microchannel-structured PVA hydrogel for improved water transport, and glycerol doping to minimize heat loss. This results in an evaporation rate of 3.26 kg m^−2^ h^−1^ under irradiation, showing promise for freshwater production from seawater and sewage. Figure 8B shows the performance of the EGaIn@Ag–PVAG hydrogel in solar-driven thermoelectric conversion (SDTC). The device, consisting of a Xenon light source, hydrogel, thermoelectric module, copper plate, and foam wool (Figure 8B(a)), generates a voltage from the temperature difference. The hydrogel’s surface temperature increased to 51.5 °C after 15 min of irradiation, stabilizing at 10 min (Figure 8B(b)). The integration of thermoelectric conversion offers a dual-functionality approach for energy harvesting, but further research is needed to optimize the device’s efficiency and stability under continuous exposure to sunlight and variable environmental conditions.

In addition to photothermal and thermoelectric applications, LM–polymer composites are also employed as Joule heaters [32]. By embedding LM within a thermoplastic polyurethane-wrapped fiber, stretchable fabrics can be created that rapidly heat up when a DC voltage is applied. While this presents a promising application for flexible heating elements, challenges related to the uniformity of heating across the fabric and the longevity of the conductive properties under repeated use need to be addressed. Furthermore, LM–hydrogel composites can be used in electroluminescent devices, where they convert electrical energy into light. The LM–hydrogel serves as the flexible top and bottom layers, while ZnS:Cu acts as the dielectric emitting layer. In one work, the LMBP hydrogel integrates LM and MXene into a double-network matrix of bacterial nanocellulose (BNC) and polyacrylic acid (PAA). Multilayered MXene (Ti_3_C_2_T_x_) is prepared via LiF/HCl etching, while BNC, modified with sulfamic acid, forms ultralong nanofibers (≈11 μm) with high electronegativity (zeta potential: −106 mV), enabling strong physical interconnections. Bulk LM and MXene are dispersed into the gel prepolymer, creating a ternary system of LM nanodroplets and MXene sheets stabilized within the BNC matrix. Sonication promotes MXene exfoliation, while hydrogen bonding with BNC prevents agglomeration. This approach yields a stable and well-dispersed hybrid hydrogel for use in electroluminescent devices. Figure 8C illustrates a three-layer hydrogel-based flexible electroluminescent device comprising an LMBP (bottom), an LBP (top), and a ZnS:Cu middle layer. Under high-frequency AC voltage, a stable electric field forms between the layers, causing the middle layer to emit bright luminescence due to electron excitation [101]. The development of flexible electroluminescent devices using LM–hydrogel composites shows exciting potential for wearable electronics, but optimizing the brightness and stability of the emitted light, particularly under different environmental conditions, is essential for future advancements.

### 6.3. LM–Hydrogel Composite in Sensor and Biomonitoring Applications

**Figure 9:**
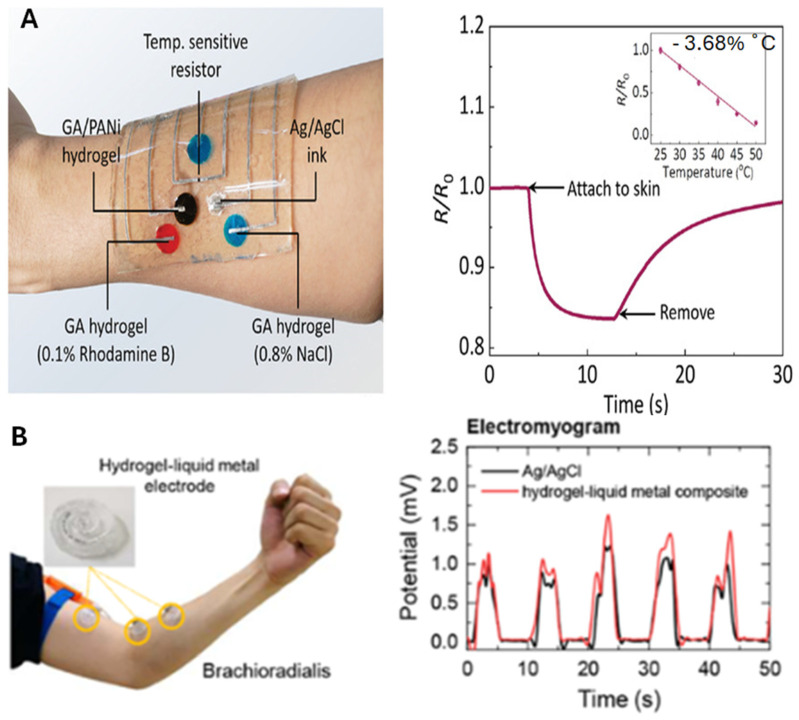
(**A**) Application of gelatin–alginate–glycerol hydrogel with patterned LM and sensing units for electronic skin, along with the resulting data. Reproduced with permission from [105], Copyright 2022, Wiley. (**B**) Electrode placement and EMG signal processing. Reproduced with permission from [78], Copyright 2020, ACS Publications.

Advances in artificial intelligence and sensing technologies have increased the demand for flexible wearable electronics, with hydrogels emerging as promising materials due to their flexibility and adhesion [102,103]. A notable strength of hydrogels lies in their inherent flexibility, which is essential for wearable sensors that must conform to the human body, but their single-network structure limits mechanical resilience. A high-performance PVA-based conductive hydrogel with CNFs, MgCl_2_, EG, and LM was developed, offering exceptional tensile strength (3.86 MPa), elongation (918.4%), and compressive strength (4.04 MPa at 80% strain). Its conductive network enables applications like micro-stress detection and Morse code transmission, making it suitable for sensors [104]. However, the main weakness is that while the tensile strength is high, the elongation at break may still be limited compared to other materials, potentially affecting its performance in more extreme applications. Sustainable hydrogel-based soft electronics were developed using a gelatin–alginate hybrid hydrogel integrated with patterned LM. This durable, transparent, and recyclable hydrogel enables multifunctional sensing for strain, temperature, heart rate (ECG), and pH. On the downside, however, the integration of LM may introduce challenges related to the uniformity of the pattern and potential material instability over time, which could affect long-term reliability. Figure 9A highlights its LM-patterned sensing units and iontophoretic drug delivery capability. The temperature sensor, using a negative temperature coefficient (NTC) resistor, shows a resistance drop of 3.68%. When applied to the forearm, resistance decreases by 23%, reflecting a 6.25 °C rise, returning to baseline after removal [105]. In another study, the LM–hydrogel composite is synthesized by dissolving 20 wt% PVA and 2 wt% agarose in deionized water, mixing with 5 wt% borax to form a gel, and removing air bubbles by compressing the gel in a syringe. After 3D printing molds to shape the hydrogel, LM is injected to create a composite electrode for biosignal detection (EMG, ECG, and EDA). A key strength of this method is the ability to 3D print the hydrogel into customized shapes, enabling precise electrode placement, which is critical for wearable sensors. However, the challenge lies in ensuring the repeatability of the printing process, as small variations in mold or LM injection can lead to inconsistencies in electrode performance. Figure 9B shows the electrode placement for EMG measurement on the arm. Signals from muscle flexion were recorded using LM–hydrogel (red) and Ag–AgCl (black) electrodes, filtered, rectified, and smoothed. The comparative merit of the LM–hydrogel electrode is its ability to produce higher-intensity peaks, indicating a stronger response signal compared to the conventional Ag–AgCl electrodes, which could improve the overall performance of biosignal detection [78]. In another study, a flexible PDMS–acrylic acid and acrylamide (pAA–AAm)–LM composite was used as prototype ECG electrodes. The flexibility of this composite is a major advantage in ensuring comfort during long-term wear, a critical factor in ECG monitoring. An ECG test was conducted to validate the performance of the LM–hydrogel electrodes in a real-world setting. A two-electrode system was used, with one electrode placed on the interior of each wrist. The results indicated that the resulting devices exhibit signal-to-noise ratios superior to those of commercial ECG electrodes [47]. The main strength of this approach is its potential to offer better performance in real-world applications, where noise reduction is essential for accurate signal detection. However, the long-term stability and biocompatibility of the LM-based electrodes may still pose concerns, particularly in continuous or long-duration usage.

### 6.4. LM–Hydrogel Composite in Biomedical Applications (Toxicity and Treatment)

**Figure 10:**
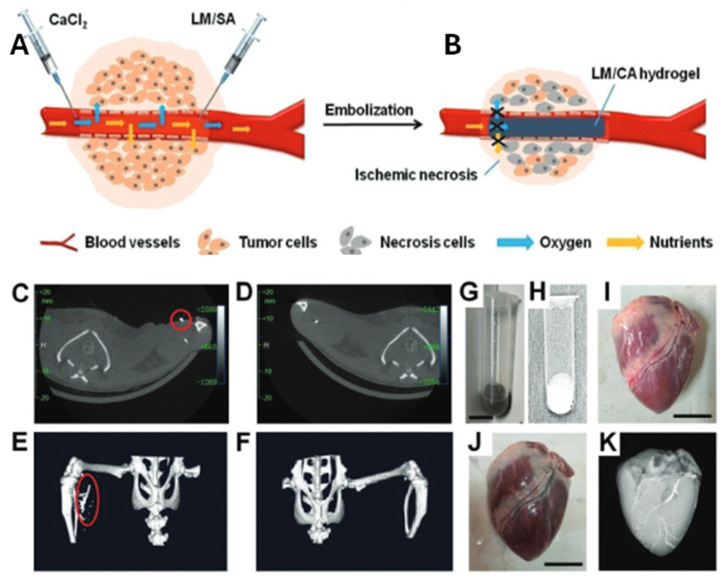
(**A**,**B**) The endovascular embolization process of the LM–CA hydrogel. Micro-CT and 3D CT images show LM–CA hydrogels in a rat femoral vein ((**C**,**E**) red circles) compared to the control (**D**,**F**). LM–SA mixture solution (LM:SA = 1:2) is shown in a photograph and X-ray ((**G**,**H**) scale bar: 1 cm) and in pig heart blood vessels before and after injection ((**I**–**K**) scale bar: 5 cm). Reproduced with permission from [108], Copyright 2020, Wiley.

Liquid metal (LM)–hydrogel composites have emerged as promising materials in biomedical applications, particularly for addressing toxicity concerns and advancing tumor treatment strategies. One major strength of these composites is their ability to leverage the unique properties of liquid metals, such as their flexibility and high surface area, which can be critical for targeted drug delivery and tumor treatment. In one study, ligand-mediated LM was developed for tumor therapy by leveraging probe sonication and the oxide layer of LM [106]. Hyperbranched poly(amido amine) (HPAA) acted as a stabilizing ligand, forming LM nanodroplets with a flaky morphology. The multivalent interactions between HPAA’s amine groups and the gallium oxide monolayer facilitated the selective adsorption of tertiary amines on gallium oxide nanocrystals. This reduced surface tension and directed nanodroplet growth along specific orientations. This approach offers the advantage of selective adsorption, enabling better control over the size and morphology of LM droplets, which is essential for drug loading and sustained release. However, the challenge with this method is ensuring the consistent and scalable synthesis of LM nanodroplets with controlled sizes and stability under physiological conditions. The LM nanoflakes efficiently loaded drug molecules such as doxorubicin (DOX) and were encapsulated in a Pluronic F-127 hydrogel for pH-responsive, sustained drug release, aiding in preventing postoperative tumor recurrence. This method demonstrates the dual functionality of the LM–hydrogel composite, serving both as a drug delivery system and a means of enhancing therapeutic efficacy. However, the long-term stability of the drug-loaded composites in vivo remains a concern, as hydrogel-based systems may degrade or lose their structural integrity over time, potentially compromising the drug delivery process. Hydrogel-based systems tend to degrade over time, which can lead to the loss of structural integrity and compromise the drug delivery process. Additionally, the long-term accumulation of metal ions from LM particles within the body could pose potential risks, including toxicity, inflammation, and immune responses. These issues necessitate extensive preclinical and clinical testing to assess the biodegradability and safe excretion of LM-based composites, ensuring that toxic buildup does not occur over time. In a separate study, a PNIPAM–LMP hydrogel was synthesized by replacing DI water with LM particle (LMP)-dispersed water during polymerization. This hydrogel demonstrated improved biocompatibility by embedding LMPs within the PNIPAM matrix, which reduced direct cell exposure to LMPs. Live/dead cell imaging of 3T3 cells cultured with 5 wt% LMPs revealed the cytotoxicity of bare LMPs, whereas PNIPAM–LMPs exhibited cell viability comparable to the control group. A major strength of this approach is its enhanced biocompatibility, which reduces toxicity while still enabling the incorporation of LM particles for their therapeutic potential. The reduced toxicity was attributed to the majority of gallium ions being incorporated into the hydrogel crosslinking and residual ions being removed during fabrication [107]. However, the long-term impact of residual gallium ions in the final composite remains a critical concern. Residual toxicity from gallium ions in repeated or prolonged exposure scenarios could still cause systemic toxicity or long-term organ damage, especially in patients with compromised organ function. Therefore, long-term safety studies, including chronic toxicity assessments, bioaccumulation studies, and immune system interactions, are required to ensure the safety of LM–hydrogel composites in clinical settings. Another study employed a rapid crosslinking approach to fabricate an LM–calcium alginate (CA) hydrogel. Ultrasonication was used to stably disperse LM droplets within sodium alginate (SA), and crosslinking was induced by injecting the mixture into a calcium chloride (CaCl_2_) solution. This process produced a hydrogel with superior mechanical properties and biocompatibility, suitable for tumor-related endovascular embolization (Figure 10A,B) [108]. However, a key concern when utilizing LM in embolization is ensuring uniform dispersion of the LM droplets in the alginate matrix. Inconsistent distribution of LM particles could result in variability in mechanical properties, potentially compromising the structural integrity and performance of the composite. More importantly, regulatory hurdles related to the approval of LM-based embolic agents need to be addressed. The presence of LM particles in embolization treatments may introduce unforeseen risks, including vascular toxicity or inflammation due to their high reactivity. Thorough preclinical and clinical evaluations are needed to confirm that the LM particles do not induce unintended damage to healthy tissue or cause long-term adverse effects in patients. Additionally, an LM–hydrogel composite served as an embolic agent for tumor treatment by blocking blood flow in tumor-associated vessels, depriving tumors of essential oxygen and nutrients [108]. The application of LM-based embolic agents in tumor treatment represents an innovative approach that could be more efficient than traditional embolization techniques. One potential limitation, however, is the precise control over the embolization process, as it requires careful tuning of the LM content to ensure effective blockage without causing unintended damage to healthy tissue. In another innovative approach, LMs were combined with metal–organic frameworks (MOFs) and alginate-based hydrogels to create a dual-functional injectable formulation. This formulation proved effective in photothermal therapy (PTT) for epidermal tumors and demonstrated combined PTT/chemotherapy efficacy for skin infections [109]. This dual-functional approach enhances therapeutic efficacy by combining multiple treatment modalities, offering a more comprehensive solution for tumor treatment. However, the integration of multiple therapeutic agents may increase the complexity of formulation, potentially leading to challenges in maintaining the stability and functionality of the composite over time. These studies highlight the versatility and efficacy of LM–hydrogel composites in biomedical applications, including drug delivery, photothermal therapy, and tumor embolization, showcasing their potential for targeted therapeutic interventions. Moreover, their multifunctionality provides various treatment options in a single system. To assess the impact of LM-to-sodium alginate (SA) ratios on radiopacity, LM, a commercial contrast agent, and LM–SA solutions with varying ratios were irradiated under X-rays, and grayscale images were analyzed. Increasing LM content enhanced radiopacity, outperforming the commercial contrast agent—making it easier to track the location and progress of the treatment during procedures such as embolization. As shown in Figure 10C, the LM–CA hydrogel filled the rat femoral vein and remained stable, yielding higher CT contrast compared to the control (Figure 10D), further supported by 3D CT reconstructions (Figure 10E,F). The radiopacity of the composite is a major advantage in clinical settings, as it allows real-time monitoring during the injection process. The injectability and radiopacity of the LM–SA solution (LM:SA = 1:2) (Figure 10G,H) were tested by injecting it into the main blood vessels of a pig heart, with clear visibility under X-ray (Figure 10J) compared to the vessels alone (Figure 10I). The filled vessels displayed distinct radiopaque imaging (Figure 10K). The ability to achieve clear X-ray visibility is a strong point of the LM–SA composite, enhancing its potential as a clinically relevant embolic agent. However, ensuring consistent injectability and radiopacity across different patient demographics or anatomical sites may present a challenge, as variations in vessel size and composition could affect the performance of the composite.

**Figure 11:**
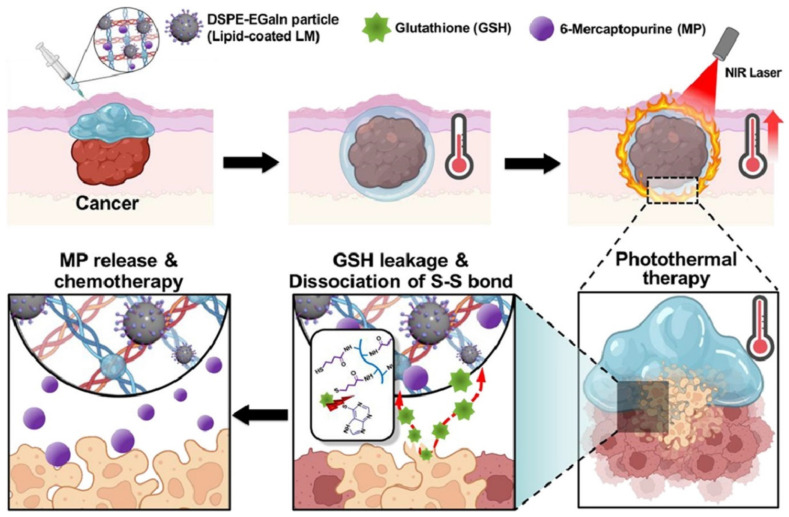
Schematic of the hydrogel composition with lipid-coated LM particles and an anticancer drug, including drug-conjugated gelatin, PEGDA, and LM particles for photothermal therapy. Reproduced with permission from [110], Copyright 2024, Elsevier.

In another study, researchers developed an injectable hydrogel platform combining LM-mediated photothermal therapy and 6-mercaptopurine (MP)-based chemotherapy for cancer treatment (Figure 11). The gallium-based LM and MP provide synergistic anticancer effects, with the hydrogel serving as a controlled delivery system. The platform induces tumor destruction through near-infrared (NIR)-mediated hyperthermia and drug release triggered by glutathione. This approach offers a promising combination of photothermal therapy and chemotherapy, capitalizing on the advantages of both modalities for enhanced therapeutic efficacy. A notable strength of this strategy is the ability to control drug release in response to the tumor microenvironment, with glutathione acting as a stimulus for targeted drug release, ensuring minimal systemic toxicity. In vitro and in vivo studies show significant tumor suppression, making this hydrogel a promising approach for the targeted treatment of solid tumors, especially triple-negative breast cancers [110]. While the concept of combining these therapeutic strategies is compelling, one limitation could be the challenge of achieving precise control over the timing and extent of drug release in vivo, as the glutathione concentration can vary in different tumor types. Additionally, the long-term stability of the hydrogel and its potential degradation products need further investigation to ensure safety and efficacy in clinical applications. These studies underline the versatility and efficacy of LM–hydrogel composites in biomedical applications, including drug delivery, photothermal therapy, and tumor embolization. However, it is essential to acknowledge the significant challenges related to long-term safety and toxicity. Addressing these concerns through comprehensive preclinical testing, long-term toxicity assessments, and regulatory compliance will be essential for ensuring the safe and effective clinical translation of these materials. The biodegradation of LM–hydrogel composites, toxicity from residual metal ions, immune system interactions, and long-term stability of the materials are critical factors that need to be thoroughly evaluated before their use in humans.

### 6.5. Wearable Triboelectric Nanogenerators (TENGs)

**Figure 12:**
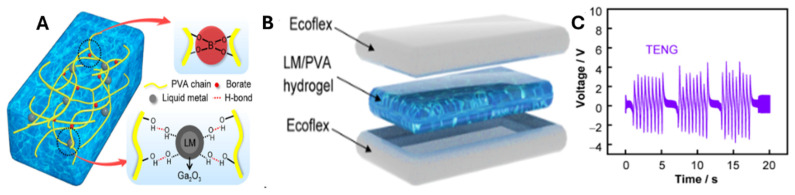
(**A**) Schematic illustration of LM–PVA hydrogel, (**B**) a schematic of LP-TENG structure, and (**C**) repeatable voltage outputs of handwriting recognition based on LP-TENG utilized for handwriting authentication. Reproduced with permission from [96], Copyright 2024, Springer.

Conventional wearable electronics struggle with reliable power supply due to the challenges of integrating heavy-duty batteries, which are limited by material and volume constraints [111,112]. Moreover, conventional energy sources, such as rigid and heavy batteries, face limitations including limited capacity, short lifespan, and safety concerns. As a result, energy supply remains a significant and challenging issue [113,114]. As a sustainable energy harvester, triboelectric nanogenerators (TENGs) convert mechanical energy from stimuli such as vibration and movement into electrical energy through contact electrification and electrostatic induction [115,116,117,118]. Hydrogels, known for their self-healing, flexibility, and biocompatibility, are ideal candidates for wearable TENG electrodes [119,120], but their use is hindered by low conductivity and poor mechanical properties [119]. To address these, researchers have added conductive fillers as discussed above. However, these fillers can cause internal stress, reducing flexibility and self-healing [121]. In contrast, gallium–indium-based liquid metal (LM) offers superior deformability, conductivity, and ease of processing [10,31,32], making it a promising conductive filler to enhance hydrogel-based TENGs [122,123,124]. For instance, in one study, an LM–polyvinyl alcohol (PVA) hydrogel-based TENG harvester (LP-TENG) (Figure 12A,B) demonstrated an open-circuit voltage of 250 V, a short-circuit current of 4 μA, and a transferred charge of 120 nC, showing high stability and outperforming most advanced TENG systems. This significant improvement in performance highlights the potential of LM-based composites to overcome the limitations of conventional hydrogels in wearable electronics. The LP-TENG was successfully applied in various multifunctional applications, including human motion detection, handwriting recognition, energy collection, message transmission, and human–machine interaction. A handwriting authentication system was fabricated using LP-TENG as the sensor and polyvinyl chloride as the pen. The LP-TENG generated distinct voltage peaks based on the pressure applied to the pen tip, capturing typical letters and words (e.g., “TENG” in Figure 12C). This system was highly sensitive, reproducible, and accurate, providing a reliable platform for information encryption and message transmission [96]. While the integration of LM in TENGs provides significant advantages, a potential weakness is the long-term stability of the liquid metal within the flexible hydrogel matrix, especially under repeated mechanical stress. The ability of LM to maintain its electrical conductivity and structural integrity during extensive use, including its resistance to leakage or oxidation over time, remains a key factor for evaluating its viability for widespread commercial use.

## 7. Summary and Future Directions

Hydrogels, with their hydrophilic and viscoelastic properties, are highly promising for flexible electronics but face challenges such as poor stability, limited stretchability, and low sensitivity. Recent advancements in integrating liquid metals (LMs), like eutectic gallium–indium alloys, into hydrogel matrices have addressed these limitations. LMs enhance conductivity, mechanical performance, and self-repairing properties, making LM–hydrogel composites ideal for applications in wearable devices, biomedical sensors, energy storage/harvesting, electromagnetic interference shielding, and soft robotics. These composites effectively combine the benefits of hydrogels’ flexibility and biocompatibility with LMs’ exceptional conductivity and ductility, positioning them as leading candidates for multifunctional, sustainable electronics.

LMs are incorporated into hydrogels in two primary ways: as continuous conductive pathways or as dispersed droplets stabilized within the polymer matrix. Such configurations offer novel functionalities, including enhanced strength, self-healing, and multi-sensing capabilities. Moreover, advancements in 3D printing and LM-initiated polymerization have expanded the design possibilities of LM–hydrogel composites, enabling applications in energy harvesting, actuators, and medical devices.

Despite their promise, several challenges remain. The long-term stability of hydrogels, particularly their susceptibility to moisture loss and freezing, compromises their mechanical integrity and conductivity. To overcome this, future research should explore novel moisture-retention strategies, such as incorporating hydrophilic polymer networks or surface modification techniques to prevent dehydration. Additionally, the development of freeze-resistant hydrogels, potentially through the introduction of anti-freeze proteins or cryoprotectants, will significantly enhance the performance of these materials in harsh environmental conditions, expanding their applicability in wearable electronics and medical devices that operate in low-temperature environments.

The long-term biocompatibility and toxicity of LM–hydrogel composites in biomedical applications are also significant concerns. A deeper understanding of the interactions between LMs, hydrogels, and biological systems is essential to ensure their safe medical use. To address this, future research should prioritize in vivo studies to evaluate the chronic biocompatibility of LM–hydrogel composites, assessing potential cytotoxicity, immunogenicity, and the impact of LM ions on surrounding tissues. Additionally, the development of biofunctionalized LMs, with surface modifications that enhance biocompatibility and minimize adverse biological reactions, will be vital for advancing their use in medical implants and drug delivery systems.

Although there is extensive literature on the application of liquid metal-based composites in energy systems, such as supercapacitors and batteries [31,32], as well as on the use of hydrogels in energy systems, i.e., supercapacitors and batteries [9,125,126,127], studies specifically focusing on LM–hydrogel composites for energy applications remain scarce. Further research in this area is highly encouraged.

Another pressing concern is LM leakage at high concentrations, which poses safety risks and undermines device reliability. The development of LM encapsulation techniques that prevent leakage, such as encapsulating the LM in protective coatings or micro-capsules, will be crucial for maintaining both the functionality and safety of these composites over time. Researchers could also explore the incorporation of ionic liquids or other non-volatile solvents to prevent LM migration under mechanical stress. Future studies should focus on improving the encapsulation of LMs within the hydrogel matrix by exploring crosslinking strategies, such as using advanced covalent bonding or self-healing mechanisms, to stabilize LM droplets. Future research should focus on addressing these challenges through innovative material design and fabrication techniques. Enhanced stability, improved interfacial interactions, and advanced printing technologies will enable the seamless integration of LM–hydrogel composites into next-generation technologies. These efforts will unlock their full potential across diverse fields, driving progress in flexible electronics, biomedical devices, and sustainable energy systems.

## Figures and Tables

**Table 1:** Liquid metal hydrogels (LMHGs) for soft electronic devices fabricated via liquid metal microfluidic techniques.

Composite	Fabrication Method	Key Properties	Applications	Reference
**EGaIn–PAM–PAA hydrogel**	Microchannel filling	Low impedance; signal-to-noise ratio: 102.6; Young’s modulus: 0.4 MPa	ECG electrodes; health monitoring devices	[47]
**EGaIn–alginate–PAM hydrogel**	Microchannel filling	Conductivity: 106 S·m^−1^; stretchability: 550%; interfacial toughness: 50 J·m^−2^	Cardiac patches; NFC; human body monitoring	[4]
**Galinstan–GelMA hydrogel**	Microchannel filling	Stable resistance change: 5% increase (50% deformation for 1000 cycles); high conductivity; response time: 51 ms; strain sensitivity: GF ≈ 47 (diameter = 200 μm); underwater sensing	Wireless human health monitoring; drug implant	[48]

## Data Availability

No new data were created or analyzed in this study.
